# High Eosinophil Rates in Patients With Right-to-Left Shunts: An Expected Role, or an Unexpected Risk?

**DOI:** 10.7759/cureus.12849

**Published:** 2021-01-22

**Authors:** Alper Karakus, Sercan Okutucu

**Affiliations:** 1 Cardiology, Besni State Hospital, Adıyaman, TUR; 2 Cardiology, Memorial Ankara Hospital, Ankara, TUR

**Keywords:** right to left shunt, patent foramen ovale, eosinophil, complete blood count, thrombosis, migraine headache

## Abstract

Background and objective

Eosinophils are associated with thromboembolic events. Since eosinophils are eliminated in the pulmonary vasculature, right-to-left shunt (RLS) through patent foramen ovale may increase eosinophils in the peripheral blood. In this report, we evaluated the eosinophils of patients with regard to the presence of RLS and its quantity.

Patients and methods

In this retrospective observational study, we analyzed the complete blood cell count (CBC) of patients with RLS (n=47) and without RLS (n=31) diagnosed by contrast echocardiography (CE). RLS was identified as mild (5-10 bubbles) and moderate shunt (10-25 bubbles).

Results

Age and CBC were not significantly different between the groups, with the exception of eosinophils. Patients with RLS had higher eosinophils percentage compared to patients without RLS (3.1 ±1.5 vs. 1.7 ±0.7, p=0.001). Additionally, eosinophils percentage was significantly higher in the mild RLS group (2.4 ±0.9 vs. 1.7 ±0.7, p=0.016) and the moderate RLS group (4.3 ±1.6 vs. 1.7 ±0.7, p=0.001) compared to normal subjects. Also, it was significantly higher in the moderate RLS group compared to the mild group (4.3 ±1.6 vs. 2.4 ±0.9, p=0.001).

Conclusions

Eosinophils percentage was higher in patients with mild and moderate RLS compared to normal individuals. Moreover, the eosinophil rate was higher in patients with moderate RLS than in patients with mild RLS.

## Introduction

Eosinophils have received much attention recently due to their potential role in activating and promoting thrombosis in acute cardiovascular events [1]. Eosinophils can produce platelet-activating factor, which induces platelet, leukocyte, and endothelial cell activation, culminating in thrombosis [2]. Additionally, serum levels of eosinophilic cationic protein can predict major adverse cardiac events following the implantation of drug-eluting stents [3]. More importantly, it has been demonstrated that eosinophils store and express tissue factors that activate the coagulation cascade via factor VII and factor X [4]. Also, tissue factor, which is abundantly expressed and stored in eosinophils, accounts for thromboembolisms in hypereosinophilic conditions [5].

Patent foramen ovale (PFO) is a residue of the physiological shunt between the right and left atrium during the fetal period. It is vital for the fetus since oxygenated blood passes to the fetal systemic circulation via PFO until birth. Incomplete fusion of the primary and secondary atrial septum generally leaves a slit-like passage or small aperture that is called PFO. Persistence and patency of PFO account for most of the systemic thromboembolism and cryptogenic strokes (CS) that are observed among young subjects and adults [6,7]. Furthermore, it may be challenging for some professionals, e.g., jet aircraft pilots, scuba divers, and long-distance flyers, due to the risk of cerebral or systemic embolism by thrombus or gas. The severity of CS or decompression illness is closely related to the presence of PFO and also the quantity of right-to-left shunt (RLS) [8,9]. Additionally, it has been reported that migraine is closely associated with RLS via PFO and clinically relieved by the closure of PFO [10]. Eosinophils have a potential role not only in thrombosis but also in histamine-related biological events such as migraine [11].

We postulated that in patients with RLS, a certain percentage of eosinophils bypass the lung tissue and increase thrombogenicity while circulating inside the cardiac chambers. To test this hypothesis, we performed this study in order to evaluate the eosinophil percentage and analyze the possible role of eosinophils in CS and migraine headaches associated with RLS.

## Materials and methods

Subjects

We retrospectively evaluated the clinical features, echocardiographic data, and complete blood cell count (CBC) parameters of 78 subjects who had undergone contrast echocardiography (CE). All subjects were male and healthy in appearance. CE was performed as a further cardiac imaging test following a high suspicion of RLS during the transthoracic echocardiography (TTE). Forty-seven patients were diagnosed as having RLS, while 31 patients were diagnosed as not having RLS on CE. Patients underwent a physical examination and their medical history was taken. All subjects were informed about the study and they signed a consent form before participation. The study was conducted according to ethical guidelines stated in the Declaration of Helsinki.

Transthoracic echocardiography

TTE was performed by an experienced cardiologist by using a commercially available system (Philips EnVisor, Andover, MA). Cardiac images were obtained using a 2.5-3.5 MHz transducer in the parasternal long-axis (PLAX), parasternal short-axis (PSAX), and apical four- (AP4C) and five-chamber (AP5C) views. The atrial septum was evaluated by color flow Doppler both at SAX and AP4C views. Standard echocardiographic parameters of the left ventricle (LV), LV internal diameter at diastole (LVIDd) and systole (LVIDs), and right ventricular diameter (RVd) at diastole were obtained by M-mode echocardiography. Additionally, LV ejection fraction (LVEF) was automatically determined by using the Teicholz formula. We measured the pulmonic (Qp) and systemic (Qs) flows by using the following equation: 

D2 (diameter) x 0.785 x velocity-time integral (VTI) x heart rate (HR): where D is the diameter of the LV outflow tract (LVOT) and the diameter of the right ventricle outflow tract (RVOT). VTI of LVOT and RVOT was measured from AP5C and SAX by using pulsed-wave (PW) Doppler echocardiography [12]. Sample volume was adjusted to 5-7 mm with wall filters set at low levels, located at LVOT and RVOT, where the diameters were measured by 2D echocardiography. Then, the Qp/Qs ratio was calculated.

Contrast echocardiography

CE was performed as a further cardiac imaging test upon a high suspicion for RLS. Vague shunt image on the atrial septum and small (<5 mm) aneurysm of the septum or flaccid septum with bi-directional mobility following Valsalva maneuvering (VM) during the TTE were the criteria for performing CE following TTE. The contrast agent was prepared by the agitation of 10 cc sterile serum saline and 1 cc air between two injectors by medical staff who were experienced in this application. During the preparation of agitated serum saline, the subjects were instructed to perform at least three consecutive VM. Then, the agitated serum was injected from the left brachial veins under the guidance of a cardiologist, while the subjects continued with the third VM. Upon observation of the bubbles’ entrance to the right atrium, subjects were instructed to release the third VM. Three consecutive Valsalva maneuvers are routinely performed. By this application, we aimed to increase the pre-load by delaying the venous return and to increase the right atrial volume and pressure transiently in order to raise the probability of RLS through PFO. RLS due to PFO was diagnosed as the passage of a certain number of microbubbles from the right to the left atrium during the four consecutive cardiac cycles just following the filling of the right atrium by microbubbles. Nevertheless, we routinely prolong the observation period up to 10 cardiac cycles in order to detect the intrapulmonary shunting. RLS was quantified as mild (5-10 bubbles) and moderate (10-25 bubbles) shunts.

Complete blood count

CBC was performed by measuring the count, volume, and percentages of cellular components of peripheral blood. CBC consisted of white blood cells (WBC) count, hematocrit (hct) and hemoglobin (Hb), mean platelet volume and platelet count, and rates of each leukocyte parameter. Components of CBC were compared according to the presence of RLS and also the quantity of RLS. Rates of neutrophils, lymphocytes, eosinophils, etc., were used in order to standardize the count of WBC components during the comparison of variables. CBC was assessed using the venous blood sample obtained after 12 hours of fasting in the morning period and performed using the Mindray Auto Hematology Analyzer-BC-6800 device (A. Menarini Diagnostics Ltd, Wokingham, UK).

Exclusion criteria

Patients with large atrial septal aneurysms or visible defects with overt left-to-right or bidirectional shunt, enlargement of the right ventricle and atrium, which was associated with RLS or atrial septal defect, and those with echocardiographic signs of increased pulmonary arterial pressure and tricuspid regurgitation (more than mild) were excluded from the study. Additionally, a previous history of atopy, allergic reactions, parasitic diseases, asthma, and chronic obstructive disease, and chronic systemic and pulmonary diseases was considered grounds for exclusion during the data gathering from medical records. Patients with hypertension, thyroid disease, anemia, cardiac valvulopathy (more than mild), ischemic heart disease, and chronic obstructive pulmonary disease were also excluded from the study.

Statistical analyses

Statistical analyses were performed using IBM SPSS Statistics version 20 (IBM, Armonk, NY). Numerical variables with a normal distribution were presented as means ±standard deviation (SD), and numerical variables with a skewed distribution were presented as medians (minimum-maximum). The normality of numerical variables was tested by the Kolmogorov-Smirnov test. For numerical variables with normal distribution, independent samples t-test and one-way analysis of variance (ANOVA) tests were used, while the Mann Whitney U test and Kruskal-Wallis test were used for the analysis of numerical variables with skewed distribution. Post hoc analysis was performed by the Tukey test for variables with homozygous variances. Two-tailed p-values below 0.05 were considered statistically significant.

## Results

Of the total 78 subjects (mean age: 25.5 ±4.5 years), 31 (39.7%) subjects (mean age: 24.9 ±3.2 years) had no RLS, while 47 (60.3%) subjects (mean age: 25.8 ±5.2 years) had RLS on CE. Of the 47 subjects with RLS, 31 had mild RLS whereas 16 had moderate RLS.

**Figure 1:**
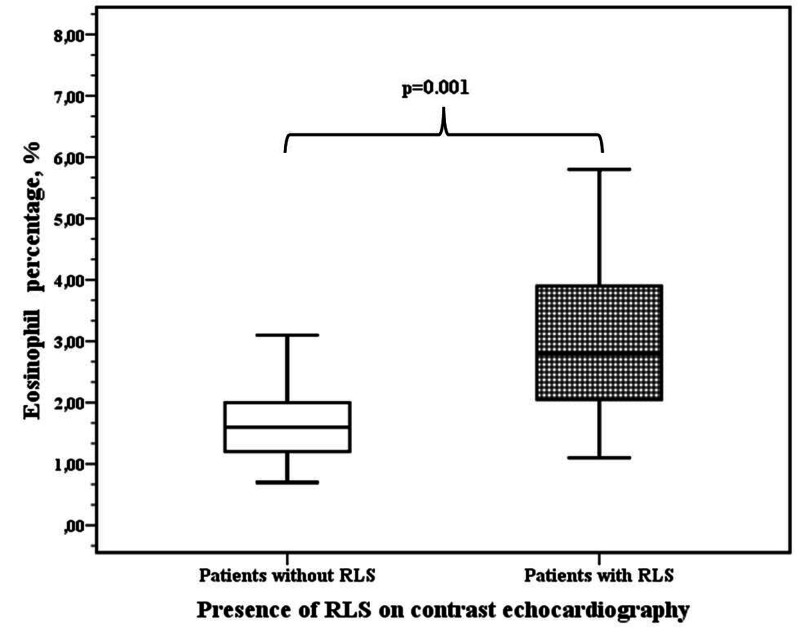
Eosinophil percentages in patients with RLS and without RLS on contrast echocardiography RLS: right-to-left shunt

The differences in age, LV and RV echocardiographic parameters, and the ratio of Qp/Qs according to the presence of RLS and its quantity were not statistically significant between the groups. However, differences in CBC parameters [WBC, Hb, and Hct; percentages of neutrophil, lymphocyte, basophil, and monocyte; and neutrophil-to-lymphocyte ratio (NLR)] according to RLS and its quantity were statistically significant. Eosinophil percentage was significantly higher in patients with RLS (3.1 ±1.5 vs. 1.7 ±0.7, p=0.001) (Table 1, Figure 1).

**Table 1:** Comparison of complete blood cell components, echocardiographic parameters of left and right ventricle, and the ratio of Qp/Qs among subjects with and without RLS on contrast echocardiography *Independent samples t-test; ^†^Mann Whitney U test RLS: right-to-left shunt; LVIDd/s: left ventricular internal diameter at diastole and systole; LVEF: left ventricular ejection fraction; RVd: right ventricular diameter; Qp/s: the ratio of pulmonary flow to systemic flow measured by echocardiography; WBC: white blood cell count; MPV: mean platelet volume; NLR: neutrophil-to-lymphocyte ratio; SD: standard deviation

Variable	Patients without RLS (n=31)	Patients with RLS (n=47)	P-value
Age in years, mean ±SD	24.9 ±3.2	25.8 ±5.2	0.372*
Echocardiographic parameters			
LVIDd, cm, median (range)	4.7 (4.1-5.5)	4.80 (4.2-5.4)	0.333^†^
LVIDs, cm, median (range)	3.1 (2.7-3.7)	3.2 (2.8-3.6)	0.410^†^
LVEF, %, median (range)	60 (55-67)	60 (54-69)	0.735^†^
RVd, cm, median (range)	1.9 (1.6-2.2)	2 (1.7-2.3)	0.131^†^
Qp/Qs, median (range)	1.028 (0.82-1.13)	1.031 (0.82-1.15)	0.326^†^
Complete blood count analysis			
WBC, x 10^3^/µL, mean, ±SD	6.5 ±1.5	6.7 ±1.2	0.517*
Hemoglobin, g/dL, mean, ±SD	16.1 ±0.9	15.7 ±1.1	0.139*
Hematocrit, %, mean, ±SD	47.9 ±2.6	47.08 ±3.3	0.212*
Platelet, x 10^3^/µL, mean, ±SD	243.1 ±50.1	253.7 ±44.8	0.329*
MPV, fL, mean, ±SD	9.6 ±1.1	9.5 ±1.04	0.730*
Neutrophil, %, mean, ±SD	57.8 ±7.0	58.9 ±6.5	0.444*
Lymphocyte, %, mean, ±SD	33.0 ±6.8	31.1 ±6.4	0.206*
Eosinophil, %, mean, ±SD	1.7 ±0.7	3.1 ±1.5	0.001*
Basophil, %, mean, ±SD	0.43 ±0.37	0.40 ±0.20	0.667*
Monocyte, %, mean, ±SD	6.8 ±1.7	6.3 ±1.2	0.118*
NLR, mean, ±SD	1.88 ±0.7	2.0 ±0.7	0.389*

**Figure 2:**
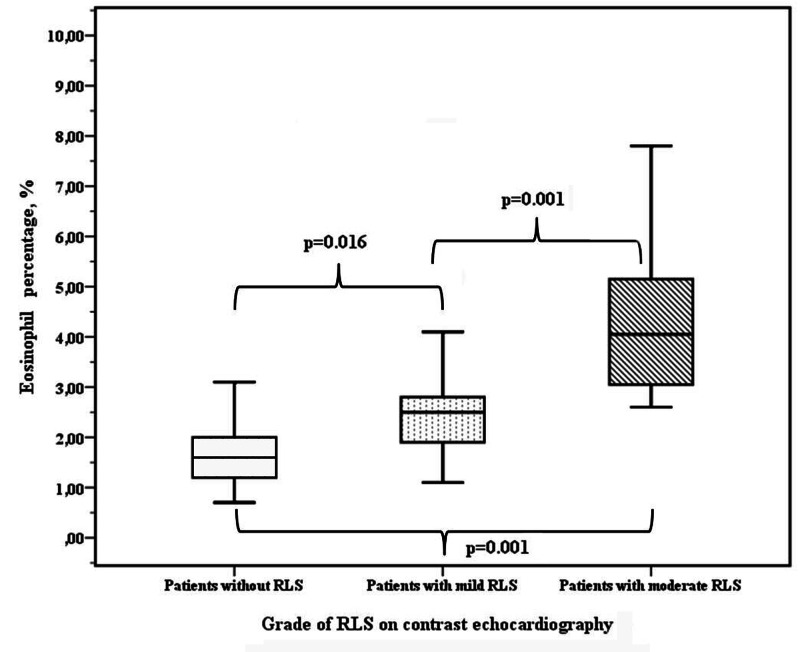
Eosinophil percentages in patients without RLS, mild RLS, and moderate RLS RLS: right-to-left shunt

Additionally, the eosinophil rate was higher in RLS subgroups compared to healthy subjects (for mild RLS: 2.4 ±0.9 vs. 1.7 ±0.7, p=0.016, and for moderate RLS: 4.3 ±1.6 vs. 1.7 ±0.7, p=0.001) (Figure 2). Moreover, the eosinophil rate was higher in patients with moderate RLS than in patients with mild RLS (4.3 ±1.6 vs. 2.4 ±0.9, p=0.001) (Table 2, Figure 2).

**Table 2:** Comparison of complete blood cell components, echocardiographic parameters of left and right ventricle, and the ratio of Qp/Qs among patients with no, mild, and moderate RLS on contrast echocardiography *One-way ANOVA test; ^†^Kruskal-Wallis test; ^§^p=0.016 for no RLS vs. mild RLS, ^‡^p=0.001 for no RLS vs. moderate RLS, ^¥^p=0.001 for mild RLS vs. moderate RLS RLS: right-to-left shunt; LVIDd/s: left ventricular internal diameter at diastole and systole; LVEF: left ventricular ejection fraction; RVd: right ventricular diameter; Qp/s: the ratio of pulmonary flow to systemic flow measured by echocardiography; WBC: white blood cell count; MPV: mean platelet volume; NLR: neutrophil-to-lymphocyte ratio; SD: standard deviation; ANOVA: analysis of variance

Variables	Subjects with no RLS (n=31)	Patients with mild RLS (n=31)	Patients with moderate RLS (n=16)	P-value
Age in years, mean ±SD	24.9 ±3.2	25.5 ±4.9	26.5 ±5.7	0.554*
Echocardiographic parameters				
LVIDd, cm, median (range)	4.7 (4.1-5.5)	4.8 (4.2-5.4)	4.8 (4.2-5.3)	0.526^†^
LVIDs, cm, median (range)	3.1 (2.7-3.7)	3.2 (2.8-3.6)	3.2 (2.8-3.6)	0.673^†^
LVEF, %, median (range)	60 (55-67)	60 (54-69)	60 (55-67)	0.893^†^
RVd, cm, median (range)	1.9 (1.6-2.2)	2.0 (1.7-2.3)	2.0 (1.8-2.3)	0.318^†^
Qp/Qs, median (range)	1.028 (0.82-1.13)	1.028 (0.82-1.13)	1.046 (0.82-1.15)	0.089^†^
Complete blood count analysis				
WBC, x 10^3^/µL, mean, ±SD	6.5 ±1.5	6.8 ±1.2	6.5 ±1.2	0.669*
Hemoglobin, g/dL, mean, ±SD	16.1 ±0.9	15.5 ±1.3	16.1 ±0.6	0.057*
Hematocrit, %, mean, ±SD	47.9 ±2.6	46.6 ±3.9	47.9 ±1.7	0.182*
Platelet, x 10^3^/µL, mean, ±SD	243.0 ±50.3	247.6 ±44.8	265.3 ±43.9	0.296*
MPV, fL, mean, ±SD	9.6 ±1.2	9.7 ±1.08	9.2 ±0.9	0.471*
Neutrophil, %, mean, ±SD	57.8 ±7.0	59.9 ±6.4	57.1 ±6.4	0.298*
Lymphocyte, %, mean, ±SD	33.1 ±6.8	30.8 ±6.7	31.6 ±5.9	0.424*
Eosinophil, %, mean, ±SD	1.7 ±0.7	2.4 ±0.9	4.3 ±1.6	0.000^§ ‡ ¥^
Basophil, %, mean, ±SD	0.43 ±0.37	0.40 ±0.22	0.39 ±0.16	0.897*
Monocyte, %, mean, ±SD	6.8 ±1.7	6.2 ±1.1	6.4 ±1.3	0.262*
NLR, mean, ±SD	1.8 ±0.7	2.0 ±0.8	1.9 ±0.5	0.458*

## Discussion

The principal findings of this study were as follows: (i) eosinophil rate in patients with RLS was higher than in subjects without RLS, and (ii) there was a more obvious eosinophil increase among patients with moderate RLS compared to ones with mild RLS and normal individuals.

Previous trials have shown that the prevalence of CS and migraine increases in patients with PFO compared to the general population. The potential association between PFO and CS has been a controversial issue for decades. Moreover, limited evidence is available on the role of PFO on the risk of CS. A meta-analysis of 134 publications also reported a higher prevalence of PFO in patients with migraine [13]. The pathophysiological mechanisms that link PFO to CS and migraine are unknown and many questions remain unanswered. Taken together, the most accepted pathophysiological hypothesis to explain the underlying relationship between PFO and CS and migraine is a microembolization, crossing the PFO and entering the systemic circulation. The powerful vasoactive and procoagulant effects of eosinophils led us to hypothesize that in patients with RLS, a certain percentage of eosinophils bypass the lung tissue and increase thrombogenicity while circulating inside the cardiac chambers.

The unrecognized role of eosinophils in thrombotic events

Uderhardt et al. have demonstrated that eosinophils have the capacity to produce strong endogenous thrombin based on the simultaneous expression of the tissue factor and the provision of a procoagulant phospholipid surface enriched with 12/15-lipoxygenase (12/15-LO) [14,15]. Furthermore, Marx et al. have highlighted the role of eosinophils in arterial thrombosis by demonstrating that eosinophil deficiency does not affect the induction time of thrombus formation and arterial occlusion [16]. It was also demonstrated that eosinophil cationic protein is an independent and predictive risk factor for thrombotic events in a large-scale epidemiological approach [14]. Furthermore, Niccoli et al. have demonstrated that enhanced activation of eosinophils at baseline is associated with thrombotic outcomes following the implantation of drug-eluting stents [3]. Considering the potential role of eosinophils in coagulation system activation and platelet activation, the findings of the present study support an association between thrombotic events and PFO, which might be of clinical relevance.

Cryptogenic stroke and patent foramen ovale

While the prevalence of PFO in the general population is approximately 25%, CS accounts for 15-40% of all ischemic strokes and occurs in approximately 50% of patients with PFO or transient ischemic attacks [17,18]. Limited evidence is available on the impact of different clinical scenarios on the risk of CS in patients with PFO. Hence, it is challenging to understand the underlying mechanism by which PFO causes a stroke. There have been a few studies focusing on eosinophils and stroke. Wang et al. investigated the diagnostic utility of eosinophil in acute ischemic stroke and showed that eosinophils might play a certain role in the occurrence, progress, and prognosis of acute cerebral infarction [19].

Migraine aura and patent foramen ovale

Danese et al. have shown that migraine with aura is more common in patients with cardiac RLS (especially PFO). The incidence of PFO is 40-60% among migraine patients with aura [20]. Of note, Lip et al. have demonstrated that migraine headache attack is associated with a higher prevalence of PFO than among the general population [21]. Interestingly, Schwerzmann et al. have demonstrated that the number of people having small RLS among migraine patients and healthy subjects was similar but a moderate or large RLS occurred more frequently in the migraine group compared to healthy controls [22]. More remarkably, it has been argued that larger PFO, persistent PFO, and complex tissue structures may cause more RLS to increase the incidence of migraine [23]. These findings may reflect the “dose-response” relationship between RLS and migraine. Several studies have suggested a link between eosinophils and neuronal homeostasis [24]. And these observations may at least partially explain the underlying pathophysiology.

As a consequence of the findings of the mentioned studies, the argument that a small embolus originating within the venous system may, hence, reach the intracranial arterial circulation to cause either stroke or migraine aura, depending on the size and location as the same pathophysiologic mechanisms, seems reasonable. Although the present study did not explore a direct role of eosinophils on CS and migraine in patients with PFO, our findings suggest that enhanced eosinophilia might contribute to the development of these conditions.

There are certain limitations that must be considered when interpreting these results. Firstly, this analysis was exploratory in nature and should be interpreted as hypothesis-generating. Secondly, the study was conducted in a single-gender arm. Another limitation is that this was a retrospective observational study. Hence, there is a need for larger and prospective studies to further investigate clinical outcomes in patient eosinophilia and PFO. However, despite the mentioned limitations, the authors expect this study will be helpful in future research on the subject.

## Conclusions

Based on our findings, the eosinophil rate in patients with RLS was higher than in subjects without RLS, Furthermore, there was a more obvious higher eosinophil trend among patients with moderate RLS compared to patients with mild RLS and normal individuals. Our results may contribute to the efforts to understand the pathophysiological mechanisms behind the increased prevalence of microembolization risk in patients with RLS.
